# Risk stratification framework to improve the utility of renal ultrasound in acute kidney injury

**DOI:** 10.4102/sajr.v28i1.2889

**Published:** 2024-07-17

**Authors:** Brendan C. Kelly, Rebecca Fung, Christopher Fung

**Affiliations:** 1 Department of Radiology and Diagnostic Imaging, Faculty of Medicine and Dentistry, University of Alberta, Edmonton, Canada; 2 Department of Medicine, School of Medicine, University College Cork, Cork, Ireland

**Keywords:** ultrasound, acute, kidney, injury, impairment, risk, factor

## Abstract

**Background:**

Acute kidney injury (AKI) is common among hospitalised patients and can lead to significant morbidity or mortality if not properly managed. Renal ultrasound (RUS) is often requested in the initial workup of AKI to rule out obstructive uropathy despite pre-renal aetiologies being implicated in most cases, especially in patients without risk factors for obstruction.

**Objectives:**

Determine the utility of RUS in detecting bilateral hydronephrosis in the context of AKI, and identify risk factors that can be used to stratify patients to better guide patient management.

**Method:**

Adults who underwent RUS for AKI between January 2019 and December 2021 were reviewed. Renal ultrasound studies that identified bilateral hydronephrosis and the patient characteristics associated with these studies were recorded.

**Results:**

Seven hundred and fifty-eight RUS reports were included. Bilateral hydronephrosis was diagnosed in 43 patients (5.7%). Of these 43 patients, 39 (90.7%) had at least one risk factor for urinary tract obstruction. Bilateral hydronephrosis was only diagnosed in 4 (9.3%) patients without any risk factor for obstruction. The risk factors with the highest odds for being diagnosed with bilateral hydronephrosis included a history of previous ureteric stenting or nephrostomy tube insertion (OR 10.37), previous bilateral hydronephrosis (OR 14.56), or multiple risk factors (OR 23.06).

**Conclusion:**

Renal ultrasound has limited utility in the evaluation of AKI in low-risk patients.

**Contribution:**

These risk factors can be used to assign patients to high- or low-risk categories to better guide management and reduce the number of unnecessary studies performed while still identifying clinically significant disease.

## Introduction

Acute kidney injury (AKI) is characterised by an abrupt impairment in kidney function that manifests as an elevation in serum creatine with decreased creatine clearance and urine output.^1^ It is a common problem encountered by clinicians, affecting up to 20% of hospitalised patients.^2^ Acute kidney injury can be secondary to both acute or chronic disease, with pre-renal aetiologies such as hypovolaemia and sepsis making up the majority of cases.^1^ Other less common aetiologies include renal pathology such as acute tubular necrosis or glomerulonephritis, and post-renal obstruction secondary to conditions such as benign prostatic hyperplasia or bladder tumours.^2^ Prompt recognition and treatment of AKI is necessary to prevent complications such as electrolyte disturbances and volume overload that can lead to significant morbidity or mortality for the patient.

Renal ultrasound (RUS) is often requested in the initial evaluation of AKI to rule out obstructive uropathy. Visualisation of hydronephrosis in this scenario can signify urinary tract obstruction; however, it is important that the ordering physician be aware of other conditions that can produce dilation of the urinary system without obstruction, and that urinary tract obstruction can be present without hydronephrosis on RUS.^3^ Distinction between bilateral and unilateral hydronephrosis is also important, as unilateral obstruction will typically only result in an elevation in serum creatinine in patients with a solitary or single functioning kidney.^4,5^

While population-based estimates of the prevalence of obstructive uropathy in AKI vary, many studies have implicated it in only 5% – 10% of AKI cases with an even lower prevalence estimated for patients without risk factors for urinary tract obstruction.^6,7^ In response to these findings, some researchers and clinicians have advocated for more selective use of RUS in AKI, with recommendations to defer or even avoid RUS in certain patient populations.^8,9,10,11^ Prior attempts have also been made at developing risk stratification frameworks for urinary tract obstruction to manage patients more effectively.^8,9,10,11^

Despite this, ordering practices have not significantly changed at our institution and, furthermore, a unifying risk stratification framework is not apparent in the literature. Therefore, the aim of this study was to evaluate the utility of RUS in the assessment of AKI, and to identify patient risk factors that can be used to categorise patients as high- or low-risk for obstructive uropathy to aid in the management of patients with AKI.

## Research methods and design

### Population

A retrospective search of an existing database of Radiology reports was conducted to identify all RUS studies performed between 01 January 2019 and 31 December 2021. The search included two tertiary care hospitals and one outpatient facility in a major city. Patients were included in the study if they were over 18 years of age and had a RUS performed in the setting of an acute impairment in renal function as defined by the requesting clinician. Studies were excluded if the patient was pregnant or post-partum, if the study was a follow-up study of previously diagnosed hydronephrosis, or if the patient had abnormal genitourinary anatomy such as a horseshoe kidney, solitary kidney or if they were a kidney transplant recipient.

### Outcomes

A total of 2108 RUS reports were obtained using this search method. Eight hundred and twenty-nine studies met the inclusion criteria. Seventy-one studies were removed based on exclusion criteria. A total of 758 RUS reports were included in the analysis. All reports were individually reviewed to identify the number of patients diagnosed with bilateral hydronephrosis and the risk factors common among this population subset. These patient characteristics were then compared to the patients in whom no bilateral hydronephrosis was observed to identify patient risk factors that could be useful for risk stratification.

### Statistical analysis

Mean patient age between the bilateral hydronephrosis and non-bilateral hydronephrosis groups was compared using a two-sample *T*-test. Patient sex between the groups was compared using a Chi-squared test. The odds ratio and 95% confidence intervals for being diagnosed with bilateral hydronephrosis for each of the identified patient risk factors were calculated and ranked. All statistical analysis was performed using IBM SPSS Statistics 28.

### Ethical considerations

Approval was obtained from the University of Alberta, Human Research Ethics Board (Pro00126337) on 09 December 2022 prior to data collection. Patient confidentiality was maintained by recording data within a password-protected file.

## Results

### Patient demographics

Demographics of patients included in the study are displayed in Table 1. The mean age of all patients in the study was 70.2 years. The mean age was not significantly different between groups with and without bilateral hydronephrosis (*p* = 0.425). The majority (55%) of patients included in the study were male, with a higher proportion of males seen in the bilateral hydronephrosis group (62.8%) compared to the non-bilateral hydronephrosis group (54.5%). There was no statistically significant difference in the proportion of male patients between groups (*p* = 0.676).

**TABLE 1:** Demographics of patients who underwent renal ultrasound for acute kidney injury.

Patient demographics	All patients (*N* = 758)			With bilateral hydronephrosis (*n* = 43)			Without bilateral hydronephrosis (*n* = 715)			*p*
	Mean ± s.d.	*n*	%	Mean ± s.d.	*n*	%	Mean ± s.d.	*n*	%	
Age (years)	70.2 ± 16.0	-	-	70.1 ± 13.0	-	-	70.4 ± 15.6	-	-	0.425
Male sex	-	417	55.0	-	27	62.8	-	390	54.5	0.676

### Bilateral hydronephrosis

Bilateral hydronephrosis was diagnosed in 43 (5.7%) patients as displayed in Table 2. Of these 43 patients, 39 (90.7%) had a risk factor predisposing them to urinary tract obstruction (Table 2). Previous urologic surgery was defined as previous transurethral resection of the prostate or bladder, or prostatectomy. Clinical signs of urinary retention included an elevated post-void residual bladder volume, palpable bladder, or urine output following insertion of catheter in a previously anuric patient. Of the 43 patients diagnosed with bilateral hydronephrosis, 4 (9.3%) did not have any known risk factors for urinary tract obstruction.

**TABLE 2:** Frequency of bilateral hydronephrosis and number of patients with high-risk medical history.

Results	*n* (*N* = 758)	%
Total bilateral hydronephrosis	43	5.7
High-risk medical history	39	5.1
Gross haematuria	1	0.1
Previous urologic surgery	1	0.1
Benign prostatic hyperplasia	4	0.5
Previous ureteric stent or nephrostomy tube	4	0.5
Previous bilateral hydronephrosis	4	0.5
Pelvic or abdominal neoplasm	4	0.5
Clinical signs of urinary retention	8	1.1
Multiple risk factors†	13	1.7

† , Defined as ≥ 2 of the above risk factors.

### Odds ratios

The odds ratio (OR) for being diagnosed with bilateral hydronephrosis for each of the identified risk factors is displayed in Table 3. All risk factors had an odds ratio greater than one. The risk factors associated with the largest increase in odds of having bilateral hydronephrosis were a history of previous ureteric stenting or nephrostomy tube insertion (OR 10.37), previous bilateral hydronephrosis (OR 14.56), and those with multiple risk factors (OR 23.06). The odds ratios for gross haematuria and previous urologic surgery risk factors were not considered significant based on confidence intervals.

**TABLE 3:** High-risk patient factors and corresponding odds ratio for having bilateral hydronephrosis with 95% confidence intervals.

Patient risk factor	Odds ratio	95% CI
Gross haematuria	1.39	0.18, 10.98
Pelvic or abdominal neoplasm	3.76	1.22, 11.58
Urinary retention	6.06	2.56, 14.34
Previous urological surgery	8.49	0.75, 95.51
Benign prostatic hyperplasia	9.63	2.71, 34.21
Previous ureteric stent or nephrostomy tube	10.37	2.91, 36.94
Previous bilateral hydronephrosis	14.56	3.76, 56.39
Multiple risk factors	23.06	9.87, 53.89

CI, confidence interval.

## Discussion

In the present study, bilateral hydronephrosis was diagnosed in only 43 of 758 patients presenting with AKI (5.7%) (Table 2). Of these 43 patients, 39 (90.7%) had at least one risk factor predisposing them to urinary tract obstruction (Table 2), and only 4 (9.3%) patients had no known risk factors. Patient age did not influence the likelihood of being diagnosed with bilateral hydronephrosis, and while more male patients were seen in the bilateral hydronephrosis group (62.8%) compared to the non-bilateral hydronephrosis group (54.5%), this difference was not statistically significant (Table 1).

The prevalence of hydronephrosis in this study is concordant with previous studies in the literature.^10,11,12,13,14^ Podoll et al. reviewed over 800 RUS performed in the setting of AKI and detected bilateral hydronephrosis in only 21 of 810 (2.6%) patients, while Liu and Wang detected bilateral hydronephrosis in 10 of 111 patients (9%) using similar methodology.^11^ A multicentre study from Spain also identified obstructive uropathy in 10% of patients presenting with renal failure.^15^ The findings from this study confirm the low prevalence of obstructive uropathy in patients with AKI, and the subsequent low pre-test probability of RUS in the initial evaluation of AKI. Most cases of AKI are attributable to pre-renal aetiologies, with studies reporting pre-renal aetiologies making up nearly three quarters of hospital cases of AKI.^16^ Additionally, among those patients that are diagnosed with obstructive uropathy, the majority have clinical manifestations of urinary tract obstruction or a history of urinary tract obstruction.^10,11^ Overall, the utility of RUS in a patient with AKI without risk factors or a clinical presentation suspicious for obstruction is very low.

Interestingly, Liu and Wang also determined urinary tract obstruction as the cause of AKI in only eight of the 10 patients with bilateral hydronephrosis.^11^ Similarly, Podoll et al. determined urinary tract obstruction as the cause of AKI in 19 of 42 patients that had either unilateral or bilateral hydronephrosis.^10^ Bilateral hydronephrosis was assumed by the authors to be physiologic or an incidental finding in these patients which is consistent with previous estimates that over 10% of patients with AKI have hydronephrosis on RUS unrelated to the cause of the AKI.^3^ The utility of RUS in AKI is even further diminished when taking this information into account as there is a significant chance that the presence of bilateral hydronephrosis may not be clinically relevant. The interpreting physician should consider this information both prior to ordering RUS and when interpreting the results of a RUS report to ensure an accurate diagnosis is made.

Previous studies have identified patient risk factors relevant to urinary tract obstruction with the goal of creating a risk stratification framework. Patient risk factors common to those diagnosed with bilateral hydronephrosis in this study included gross haematuria, benign prostatic hyperplasia, previous urologic surgery, previous ureteric stenting or nephrostomy tube placement, previous bilateral hydronephrosis, known pelvic or abdominal neoplasm, urinary retention, or multiple of these named risk factors. Odds ratios for being diagnosed with bilateral hydronephrosis were greater than one for all risk factors identified, with previous stenting or nephrostomy tube (OR 10.37), previous bilateral hydronephrosis (14.56), and multiple risk factors (23.06) being the risk factors associated with the greatest increase in odds (Table 3). Similarly, patients with a history of cancer, pelvic mass or prior renal or pelvic surgery, or history of neurogenic bladder were identified to be more likely to have hydronephrosis in a study performed by Gamss et al.^17^ Podoll et al. identified additional risk factors predisposing to urinary tract obstruction including age of 65 years or greater, history of nephrolithiasis or abdominal trauma, or known urinary tract anomaly.^10^ Other risk factors identified by Licurse et al. included frequency of urinary tract infection, known benign prostatic hyperplasia, ethnic factors along with no history of congestive heart failure, drug toxicity or pre-renal AKI.^12^ Overall, the risk factors identified in the present study overlap with those previously described, apart from gross haematuria and clinical signs of obstruction which were unique to this study. These additional risk factors can be considered by clinicians when gauging the probability of obstructive uropathy as the sole cause of AKI.

A risk stratification framework has been proposed by Licurse et al.^12^ that focused on 7 variables: (1) history of hydronephrosis; (2) recurrent urinary tract infections; (3) diagnosis consistent with possible obstruction; (4) ethnic factors; and absence of the following: (5) exposure to nephrotoxic medications; (6) congestive heart failure; (7) pre-renal AKI. Each variable was associated with a score that could be assigned to the patient to classify them into low, medium or high-risk categories. Ultimately, while the evaluation of AKI is nuanced, patients in the high-risk category, according to this model, should undergo RUS, while those determined to be low-risk, should not. Decisions for medium-risk patients are more difficult and may require additional considerations by the clinician. This study contributes additional risk factors that can be considered in this scenario with accompanying odds ratio that provides information on which risk factors may be the most important for urinary tract obstruction.

**FIGURE 1:**
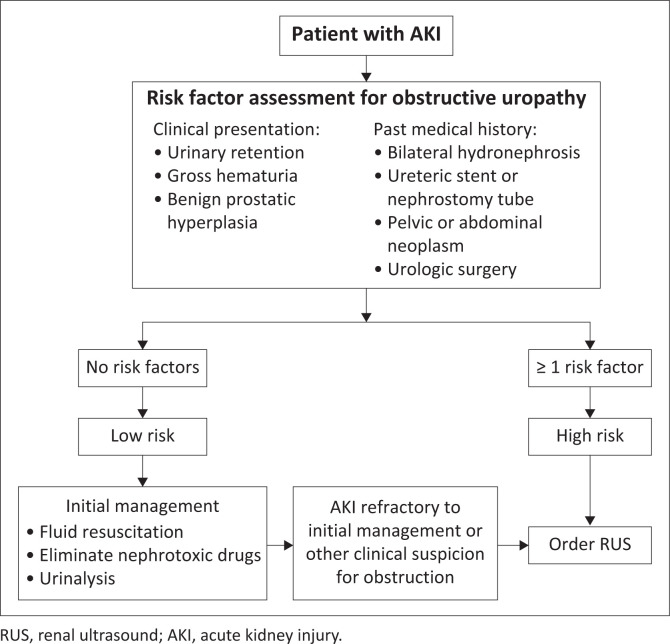
Risk stratification and clinical decision-making tool for guiding the use of renal ultrasound in the initial evaluation of patients presenting with acute kidney injury.

Given the uncertainty surrounding medium-risk categories, the authors recommend categorising patients as low-risk if they have no risk factors, or high-risk if they have one or more risk factors (Figure 1). Patients with known anatomic abnormalities such as a horseshoe kidney, solitary kidney, single functioning kidney or kidney transplant are considered high-risk and should have RUS performed given the increased risk of obstruction and severity of consequences if obstruction is not promptly identified. The authors’ recommendation is that in low-risk patients, RUS should be deferred until the patient is fluid resuscitated, nephrotoxic medications are eliminated, and urinalysis is completed (Figure 1). If kidney impairment persists or worsens despite these initial measures, or if obstruction is suspected for any other reason, RUS should be performed. Adoption of this decision-making process can likely reduce the number of unnecessary studies performed while still identifying clinically significant disease.

The distinction between unilateral and bilateral hydronephrosis was made in this study given that acute unilateral obstruction rarely results in AKI unless the patient has a single functioning or solitary kidney.^4,5^ This scenario is uncommon, although it is possible that cases of clinically relevant unilateral obstruction were excluded from the authors’ analysis. Albeit, this scenario is unlikely, and while these patients may have RUS delayed based on the presented recommendations, if renal dysfunction persists after a short trial of fluid resuscitation, RUS would still be requested to rule out obstructive pathology. Timelines regarding reversibility of post-renal AKI vary; however, differences of 24 h are likely insignificant.^18^

Limitations of this study that should be acknowledged are that the study population was limited to patients among two tertiary hospitals and one outpatient facility in a single Canadian city, potentially introducing patient bias. In addition, patients were defined as having AKI based on the history or indication provided by the clinicians as opposed to biochemical results or urine output. Taking this into consideration, the prevalence of AKI and bilateral hydronephrosis is likely even lower than estimated given that AKI is not always diagnosed according to explicit diagnostic criteria in daily practice.

## Conclusion

While RUS is commonly ordered in the initial evaluation of AKI, the prevalence of obstructive uropathy is low, especially for patients with no known risk factors for urinary tract obstruction. Risk factors identified in this study can be used by clinicians to assign patients to high- or low-risk categories to aid in the clinical decision-making process. By delaying RUS for low-risk patients until other more common causes of AKI are excluded, the number of unnecessary studies can be reduced while still identifying clinically significant disease.
